# Non-Linear Regression Modelling to Estimate the Global Warming Potential of a Newspaper

**DOI:** 10.3390/e22050590

**Published:** 2020-05-25

**Authors:** Alexis Lozano, Pedro Cabrera, Ana M. Blanco-Marigorta

**Affiliations:** 1 Department of Civil Engineering, University of Las Palmas de Gran Canaria, Campus de Tafira s/n, 35017 Las Palmas de Gran Canaria, Spain; alexis.lozano@ulpgc.es; 2 Department of Mechanical Engineering, University of Las Palmas de Gran Canaria, Campus de Tafira s/n, 35017 Las Palmas de Gran Canaria, Spain; 3 Department of Process Engineering, University of Las Palmas de Gran Canaria, Campus de Tafira s/n, 35017 Las Palmas de Gran Canaria, Spain; anamaria.blanco@ulpgc.es

**Keywords:** ecolabeling, life cycle assessment, correlation, non-linear regression models

## Abstract

Technological innovations are not enough by themselves to achieve social and environmental sustainability in companies. Sustainable development aims to determine the environmental impact of a product and the hidden price of products and services through the concept of radical transparency. This means that companies should show and disclose the impact on the environment of any good or service. This way, the consumer can choose in a transparent manner, not only for the price. The use of the eco-label as a European eco-label, which bases its criteria on life cycle assessment, could provide an indicator of corporate social responsibility for a given product. However, it does not give a full guarantee that the product was obtained in a sustainable manner. The aim of this work is to provide a way of calculating the value of the environmental impacts of an industrial product, under different operating conditions, so that each company can provide detailed information on the impacts of its products, information that can form part of its "green product sheet". As a case study, the daily production of a newspaper, printed by coldset, has been chosen. Each process involved in production was configured with raw material and energy consumption information from production plants, manufacturer data and existing databases. Four non-linear regression models have been trained to estimate the impact of a newspaper’s circulation from five input variables (pages, grammage, height, paper type, and print run) with 5508 data samples each. These non-linear regression models were trained using the Levenberg–Marquardt nonlinear least squares algorithm. The mean absolute percentage errors (MAPE) obtained by all the non-linear regression models tested were less than 5%. Through the proposed correlations, it is possible to obtain a score that reports on the impact of the product for different operating conditions and several types of raw materials. Ecolabelling can be further developed by incorporating a scoring system for the impact caused by the product or process, using a standardised impact methodology.

## 1. Introduction

Life cycle assessment (LCA) is a holistic method to account for the environmental effects of all the aspects of resource use from the extraction of raw materials to the final disposal of a product [1]. It is a powerful tool that makes it possible to calculate the environmental impacts generated by the manufacture of products or services [2]. Through the information provided by an LCA, it is possible to know the hidden costs of the products. According to the concept of radical transparency, developed by Goleman, companies committed to the environment can offer information on the impacts of their activity to their consumers [3].

An LCA study is composed of four stages [4]: a first stage, in which goal and scope aims are defined to establish the end of the assessment; a second stage, which consists of an inventory analysis based on the description of the material and energy flows within the product system and interactions with the environment, the consumed raw materials and the emissions; a third stage, in which details from inventory analysis serve for the impact assessment; and, finally, a fourth stage, based on the interpretation of the LCA, the determination of data sensitivity and the presentation of results.

Under this framework, several studies have been carried out to find correlations between impacts, greenhouse gas emissions and types or characteristics of resources used. In this sense, Berger and Finkbeiner conducted an analysis of correlations in the evaluation of impacts to measure the use of resources [5]. Results of the evaluation of the impacts were analysed by means of several indicators in order to verify if different indices lead to similar results with the aim of reducing the number of indicators. The results revealed linear regressions between the indicators that evaluate the consumption of raw materials. However, they did not display those correlations between the indices that evaluate emissions in natural resources. Park and Seo carried out evaluation of the approximate life cycle of products using the analysis of multiple regression and artificial neural networks [6]. A methodology is explored where the products are grouped according to their environmental characteristics, relating them to an environmental impact index. Based on a neural network approach, a prediction of the impacts for a certain conceptual design is made. Menten et al. made a review of LCA studies for greenhouse emissions in biofuels where a meta-regression analysis is carried out [7]. As a result of the study, a relationship between different types of fuels is revealed. Wei et al. developed a calculation model in relation to life cycle inventories and impacts, and they studied the robustness of the tool through a sensitivity analysis [8]. Grant et al. studied the use of statistical inference, especially multivariate correlation and regression, as a means of interpreting life cycle assessments [9]. Some of the main market life cycle analysis tools [10] already offer the possibility for customers to use different scenarios to calculate the impacts of products under different operating conditions.

Related to the case study of this paper, the global warming potential of a newspaper, several references highlight the importance of the environmental impact assessment of a newspaper. For example, Moberg et al. [11] addressed the potential environmental impacts of two product systems; printed on paper and electronic paper tablets. They found that the environmental impact of newspaper consumption could be reduced by the use of tablet e-paper. Dahlbo et al. [12] reported an analysis of newspaper waste management alternatives for the Helsinki Metropolitan Area. They combined a life cycle impact assessment with a social life cycle costs approach and justified the focus on a newspaper by three reasons: “(1) paper is one of the largest fractions of municipal solid waste, and waste management solutions for paper have impacts on the whole waste management system; (2) both material recycling and energy recovery are potential waste management solutions for discarded newspapers; and (3) newspaper is a fibre product derived from forests, Finland’s most important renewable natural resource.” As a result of this analysis, the authors mentioned that both, environmental and economic impacts are crucial for making sustainable decisions in the newspaper waste management area. However, the two evaluation approaches they used revealed opposite results: while an economics-focused approach seemed to lead to the worst environmental alternatives, the best environmental solution resulted in the highest costs. Another interesting and related experience was recently carried out by Liu et al. [13]. In this study, the authors integrated both material flow analysis (to determine the flow of wastepaper) and LCA, in order to construct a benchmark model of China’s wastepaper recycling decision system. The model was created by sensitivity analysis of the relevant parameters affecting the efficiency of the wastepaper recycling system. Results showed benefits for China’s wastepaper recycling in both economic and greenhouse gas emissions structure.

In this work, a new approach, based on non-linear estimators, is applied to the evaluation of the environmental impact of the different processes involved in the daily production of a newspaper. The source data for this work were obtained through a life cycle assessment (LCA) published by our research group in a previous paper [11]. In this former article, the LCA of the production process of a newspaper, printed by coldset and taking real inventory data from a plant in operation on the island of Gran Canaria (Spain), was presented.

The aim is to obtain the environmental impacts for different situations from correlations and inferences that make it possible to know the impacts, grouped or not, of any combination of scenarios without the need for specific calculation software. This methodology could be applied to almost any company and any product, so that, from this tool, companies could easily calculate the product environmental impacts, disaggregated by each type of product or product specification that the company can manufacture. 

As a general expected contribution, it is worth highlighting the possibility that will be provided to companies, with this simplified methodology, to obtain in a personalized way the environmental impacts of their products. The information required by this methodology can be updated and adapted according to the constantly changing production conditions. This can mean a definitive advance in the process of eco-labelling products.

## 2. Materials and Methods

This section describes the production process of the newspaper under study, the characteristics of the data used to estimate the environmental impact, and the regression techniques used for this purpose. 

### 2.1. System Definition and Baseline LCA Model

**Figure 1:**
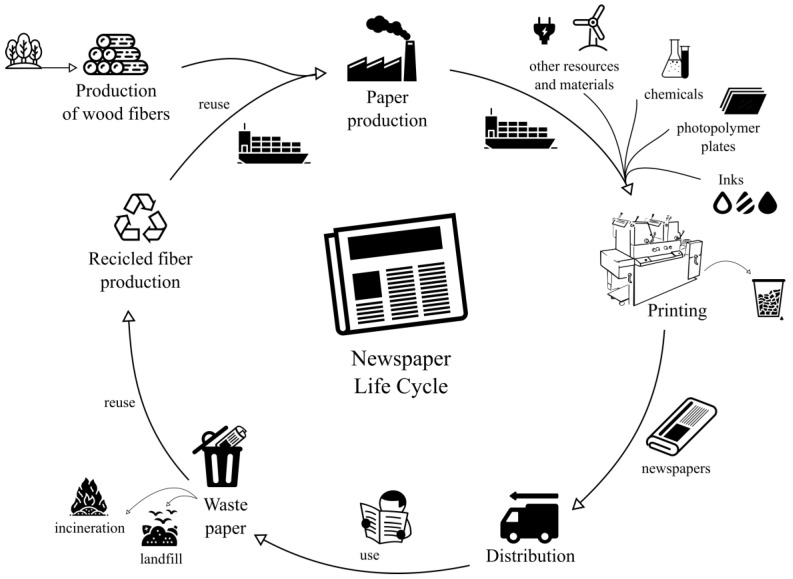
Life cycle of a printed newspaper. Figure inspired in [14].

The production process of a printed newspaper from cradle to grave, considering paper recycling, is shown in Figure 1.

The cycle begins with the production of fresh and recycled fibres needed for papermaking: the paper can be obtained either from virgin fibers of wood or from fibers recycled from the waste of newspapers and magazines. The paper is transported to the printing plant by various means of transport such as train, truck and sea. The printing centre receives the rest of the raw materials necessary for printing the newspaper: plates, inks, chemicals, ribbons, etc., as well as supplies such as electricity, water, etc. The printed newspaper is distributed to points of consumption using various means of transport, usually road and air. It is a perishable product that expires during the day; part of the daily production is recycled, and the rest is incinerated or wasted in landfills.

In our previous article [14], life cycle analysis of the production process of a newspaper is presented, taking real inventory data from a plant in operation on the island of Gran Canaria (Spain). The product system of this study is “printed newspaper by coldset technology”. System boundaries cover different stages of the production process that are grouped into the following process units:

1. Prepress: platemaking processes, processing, folded, and perforated plates.
2. Printing: the entire supply process (paper, chemicals, ink…), maintenance, and cleaning of the printing machine.
3. Finishing: transportation of the printed product, stacking, packing, and delivery.
4. Distribution.

**Figure 2:**
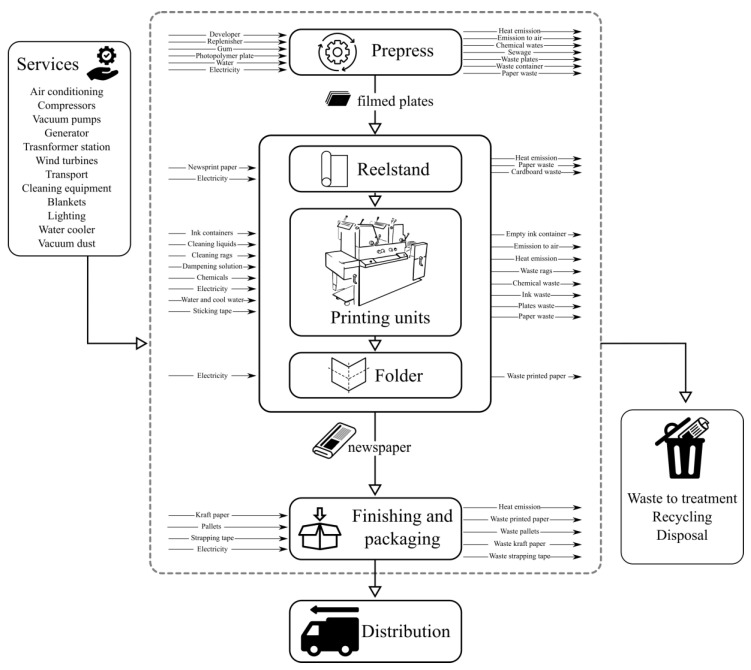
Flowchart of the main process units.

Figure 2 shows a detailed flowchart of the main process units and their intermediate processes. The general supplies have been grouped under the name “services”. The waste generated by the different units requires specific treatment and has been grouped under the name “recycling”.

Various operating, manufacturing and distribution conditions are analysed in this previous work [14]. As a baseline scenario, a 64-page newspaper in a tabloid format of 390 × 289 mm with a circulation of 10,000 copies and newsprint of 45 g/m^2^ is considered. As functional units, both FU-1: per kg of paper and FU-2: per unit of newspaper, are used. Both functional units are related by: FU-2 = FU-1 × (weight × page surface × number of pages/2). Other scenarios consider different product specifications: run (copies), weight (gr/m^2^), size, location and technology of the paper factory, location of the printing plant, distribution locations, and electricity source and mix. The methodology ReCiPe [15,16] is used in order to calculate the midpoint impacts [17] for different categories of damage: human health, ecosystems, and cost of resources [12,18]. As a result, the article shows the environmental impacts for each of the scenarios.

### 2.2. Data Used in the Model

The data used in this paper have been extracted from the results presented by Lozano-Medina et al. [11]. The compilation includes the environmental impacts generated in the life cycle of a printed newspaper considering different possible scenarios. These scenarios involve manufacturing specifications (page, grammage and height) and characteristics of the paper as main raw material, in terms of composition, origin and manufacturing process, and number of printed copies. Thus, the following variables have been taken:

- Number of pages: On the one hand, in the newspaper used for this study, the number of pages that can be printed simultaneously varies by multiples of 16. On the other hand, the minimum number of pages that a newspaper usually has, which corresponds to the sports type, is 32 pages and the maximum is 64. In our model, we have therefore considered 32-, 48- and 64-page newspapers.
- Grammage: The grammage represents the mass of paper per printed surface. Newspapers and magazines on high-quality newsprint weigh more than 60 g/m^2^. In the case of sports newspapers and daily newspapers, the usual weight is 45 g/m^2^. In the model, we have considered weights of 42, 45 and 48.8 g/m^2^.
- Height: The surface of the paper that forms a newspaper page is delimited by the width, which depends on the development of the printing rollers and which is usually a fixed parameter; in our case it has a value of 289 mm. The height varies according to the length of the paper rolls used. Three different paper sizes have been considered, resulting in heights of 360, 390 and 420 mm.
- Paper type: The "type of paper used" parameter includes several variables:
  - The location of the paper mill, which is related to the local energy mix. In areas of northern Europe and Canada, there is a large amount of hydroelectric power; in central Spain, the energy mix contains mainly fossil fuels.
  - The transport to the printing plant, which is usually by train, truck and ship. It is necessary to consider the kilometres covered by each means.
  - The printing technology and the raw materials are recycled in different percentages.

The different combinations of paper, taking into account its composition, the manufacturing process, the origin of materials, and the technology, are as follows:

1. Madrid: 100% recycled deinked pulp.
2. Belgium 100% recycled deinked pulp.
3. Sweden: 50% recycled deinked pulp.
4. Canada: 0% recycled deinked pulp.

- Print run (number of copies): The number of copies to be printed is a parameter that varies depending on the print run requested by each publisher. In the current market, print runs are decreasing because the reading of printed paper is being replaced by reading through digital devices. Printing technology and new machines have been adapted to produce saleable copies with very low print runs. The machine under consideration may have saleable copies from 250 invalid copies. A newspaper has fixed costs for capital, labour, and printing plates that are distributed among the printed copies, so that the unit cost and impacts of a newspaper decrease as the circulation increases. We have considered variations in print runs between 500 and 50,000 copies.

Table 1 shows an extract of all the combinations of parameters and data used as input data in the regression functions, to carry out the training, validation and testing of the models. As environmental impact parameter, the climate change impact in terms of kg CO_2_ eq., was used.

**Figure 3:**
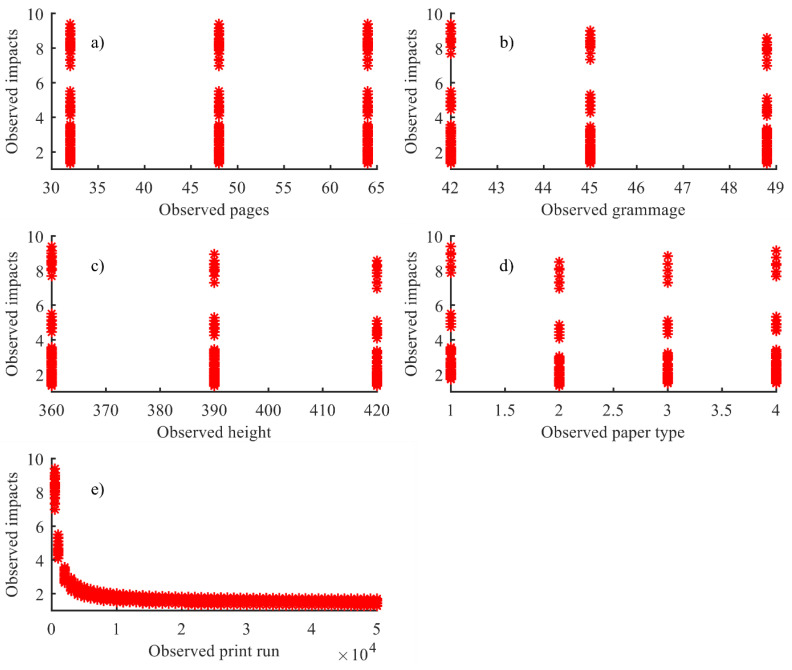
Representation of each individual variable against the impact. Individual behaviour of impacts vs. (**a**) number of pages; (**b**) grammage; (**c**) heights (**d**) paper types and (**e**) print run.

Figure 3 shows graphically the individual behaviour of every independent variable considered and the target variable (environmental impact of the newspaper).

### 2.3. The Multiple Regression Functions

As described before, four variations of a non-linear regression model [19] have been analysed to estimate the impact of a newspaper’s circulation.

The decision to consider non-linear regressions to model the environmental impact of this product, rather than a linear procedure, was taken on the basis of two main criteria. Firstly, the non-linear approach is a more general procedure than a linear regression [19]. Since linear regression is a special case of nonlinear regression, a non-linear function can fit any model, including a linear one [20]. With an adequate estimation of the parameters that define a non-linear function, it is possible to obtain a final linear model. This happens when the non-linear terms of the function are nulled, or notably reduced, because of the small value obtained by its corresponding estimated parameters. Another reason is a more specific criterion related to the behaviour of the different variables involved in the production process of the analysed product. As can be shown in Figure 3, the first four variables (number of pages, grammage height, and paper type) have non-linear behaviour with respect to the response variable (environmental impacts). Just the fifth variable (print run) shows a behaviour which can be modelled by a linear function. Additionally, a practical criterion has conditioned the decision of using non-linear regressions as an alternative to other more elaborate and complex algorithms used in the literature [6,7,8,9,10]. With the procedure carried out in this paper, a simple regular expression is obtained. Thus, companies in the sector are provided with a simple tool with which they can easily obtain the environmental impacts of their products.

The impact assessments performed by the four non-linear models are based on the adjustment and subsequent use of the multiple regression function defined by Equation (1).

(1) $$y_{i}=f(x_{1},x_{2},\ldots ,x_{k};\beta )+\varepsilon_{i},\;i=1,\ldots ,n$$

where $y_{i}$ is an observation of the variable response (or dependent variable); $x_{i}={\left(x_{1},x_{2},\ldots ,x_{k}\right)}^{T}$ is an observation of the each k input variables, usually called explanatory, regressor, or independent variables; *ε_i_* is the random noise of each observation of the response variable; and $\beta ={\left(\beta_{1},\beta_{2},\ldots ,\beta_{p}\right)}^{T}$ are the p coefficients, or parameters, which define the relationship between the input variables and response variable in function f.

Therefore, to be able to approximate the response variable and to obtain new estimations from input variables, is necessary to obtain a function $f(x_{};\beta )\;$, based on a given training set $D=\left\{\left(x_{i},y_{i}\right),i=1,\ldots ,n\right\}$ with *n* samples of each variable.

A brief description of the models used is presented in the following section and more detailed description of the non-linear regression foundations can be found in [19].

#### 2.3.1. Description of Non-Linear Regression Models Used

The non-linear regression models considered in this work follow the original formulation represented by Equation (2),

(2) $$y_{i}=\beta_{1}+\beta_{2}x_{1}{}^{\beta_{3}}+\beta_{4}x_{2}{}^{\beta_{5}}+\beta_{6}x_{3}{}^{\beta_{7}}+\beta_{8}x_{4}{}^{\beta_{9}}+\beta_{10}x_{5}{}^{\beta_{11}}+\varepsilon_{i},\;i=1,\ldots ,n$$

where $\beta ={\left(\beta_{1},\beta_{2},\ldots ,\beta_{p}\right)}^{T}$ are the unknown parameters to find for the adjustment of the function $f(x_{};\beta )$. This first original model has been varied three times to obtain a total of four non-linear regression expressions, defined by Equations (2)–(5).

(3) $$y_{i}=\beta_{1}+\beta_{2}x_{1}{}^{\beta_{3}}+\beta_{4}x_{2}{}^{\beta_{5}}+\beta_{6}x_{3}{}^{\beta_{7}}+\beta_{8}x_{4}{}^{}+\beta_{9}x_{4}{}^{\beta_{10}}+\beta_{11}x_{5}+\beta_{12}x_{5}{}^{\beta_{13}}+\varepsilon_{i},\;i=1,\ldots ,n$$

(4) $$y_{i}=\beta_{1}+\beta_{2}x_{4}{}^{}+\beta_{3}x_{4}{}^{\beta_{4}}+\beta_{5}x_{5}{}^{{}_{}}+\beta_{6}x_{5}{}^{\beta_{7}}+\varepsilon_{i},\;i=1,\ldots ,n$$

(5) $$y_{i}=\beta_{1}+\beta_{2}x_{4}{}^{}+\beta_{3}x_{4}{}^{\beta_{4}}+\beta_{5}x_{5}{}^{{}_{\beta_{6}}}+\varepsilon_{i},\;i=1,\ldots ,n$$

#### 2.3.2. Algorithm for the Estimation of β Unknown Parameters

The estimation of the ***β*** unknown parameters is carried out with the Levenberg–Marquard (LM) Algorithm. This is a hybrid optimization technique that uses both Gauss–Newton and steepest descent approaches to converge to an optimal solution [21]. It takes advantage of the high speed of the Gauss–Newton algorithm and the high stability of the steepest descent method [22]. In this work, the algorithm finds the best set of unknown parameters in order to minimize error between the response variable (environmental impacts obtained from the non-linear functions) and the actual values (actual observations of the response). Basically, the LM algorithm provides a numerical solution to the problem of minimizing a nonlinear function, over a space of parameters for the function (***β***). 

### 2.4. Metrics Used to Evaluate Numerical Estimations of the Models

The metrics used in this paper to evaluate the numerical estimations of the proposed models were mean absolute error (MAE), mean absolute percentage error (MAPE), and R-squared.

*MAE* is defined by Equation (6) where the *n* estimated values are represented by the letter “*e*” and the *n* observed values by the letter “*o*”. MAE is expressed in the same units as the parameters it compares [23].

(6) $$MAE=\frac{1}{n}\sum_{i=1}^{n}\left|o_{i}-{\hat{e}}_{i}\right|$$

*MAPE* is defined by Equation (7) and is a relative measurement that expresses the error as a percentage of the observed data [23].

(7) $$MAPE=\frac{100}{n}\sum_{i=1}^{n}\left|\frac{o_{i}-{\hat{e}}_{i}}{o_{i}}\right|$$

R-squared is defined by Equation (8) and indicates the proportionate amount of variation in the response variable, *y*, explained by the independent variables, *x* [6]. *Although some references in literature have warned against the use of this index in a non-linear context [24,25,26], we have decided to include it, and to analyse it with caution, because it is a very common and intuitive metric.

(8) $$R^{2}=\frac{SSR}{SST}=1-\frac{SSE}{SST}$$

*SSE* is the sum of squared errors, *SSR* is the sum of squared regression and *SST* is the sum of squared totals. 

### 2.5. Method for the Training and Testing of the Four Models Analysed

**Figure 4:**
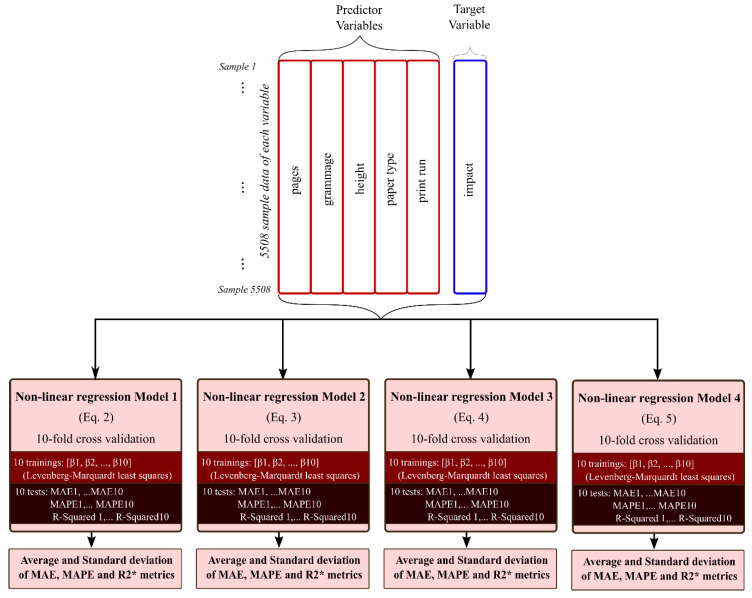
Method developed for testing each analysed model.

In Figure 4 is shown a schematic representation of the procedure used to evaluate the four non-linear models used in this study. As can be observed in the upper part of the figure, from the whole dataset collected for the study, predictor variables (inputs to the models) and target variables (output of the model) are distinguished. Then, the 10-fold cross-validation technique [27] is used to train and test each model ten times. 

**Figure 5:**
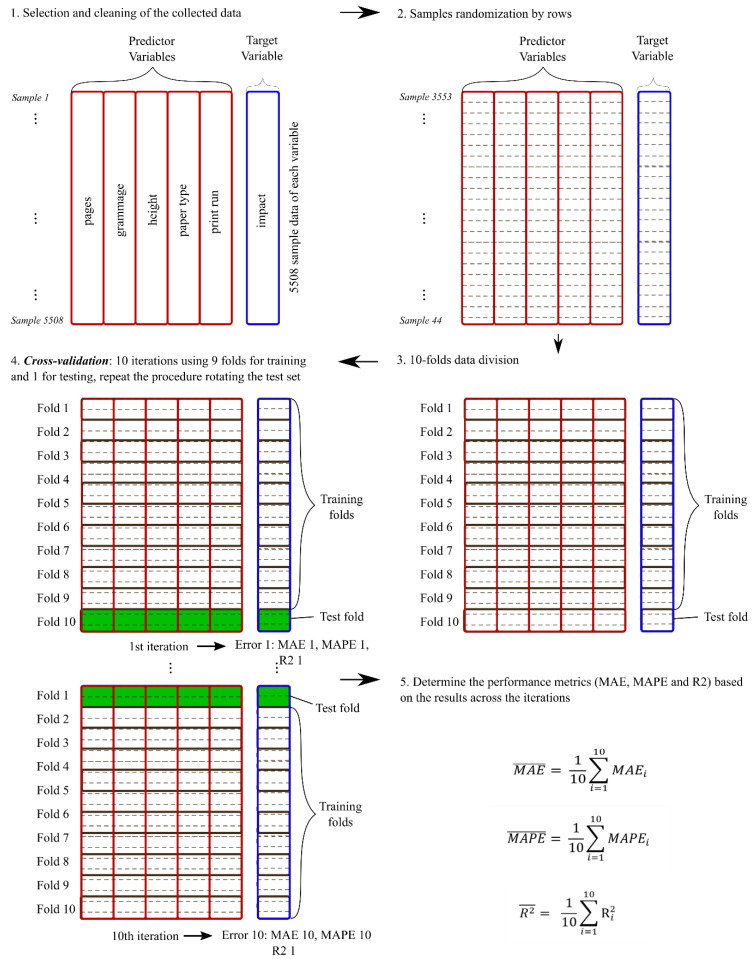
10-fold cross-validation applied to evaluate each model. (1) Data selection and cleaning; (2) data randomization by rows; (3) data division; (4) Cross-Validation; (5) Performance determination by metrics.

In Figure 5 is shown a more extensive description of the 10-fold cross-validation application. It follows the following steps:

1. Desired variables are selected, and missing values are checked to delete all the sample rows (if missing data exist in some variable of the observation).
2. Samples are randomized by rows, to guarantee the data is representative.
3. The whole dataset is divided in 10 parts, or folds, of the same dimension (each one with the same number of samples).
4. Nine data folds (90% of the total dataset samples) are considered a temporal training dataset and they are used to train the model. The remaining fold (10% of samples) is considered a temporal data test set and it is used to carry out the testing of the model.
5. With the training dataset, the models defined by Equations (2)–(5) are adjusted and unknown **β** parameters are estimated using the Levenberg–Marquardt nonlinear least squares algorithm [19].
6. This procedure is repeated 10 times for each model, to rotate the test dataset and obtain 10 evaluations in each case. As results, 10 values of error, calculated following a statistic metric formulation, are obtained. The statistical metrics considered measure the error between the true value of the response and the estimated value obtained by the model. In this study, the error metrics considered were MAE, MAPE and R-squared.
7. Finally, the average mean values of the 10 values obtained for each metric and standard deviation are calculated.

Therefore, after applying the overall procedure defined in Figure 4 and the 10-folds cross-Validation technique defined by Figure 5, an average mean and a standard deviation of 10-MAE, 10-MAPE and 10-R-squared measures are obtained for each tested model (see Table 2, Table 3 and Table 4).

(9) $$\bar{MAE}=\frac{1}{10}\overset{10}{\underset{i=1}{\sum }}MAE_{i}$$

(10) $$\bar{MAPE}=\frac{1}{10}\overset{10}{\underset{i=1}{\sum }}MAPE_{i}$$

(11) $$\bar{R^{2}}=\frac{1}{10}\overset{10}{\underset{i=1}{\sum }}R_{i}^{2}$$

## 3. Results

**Figure 6:**
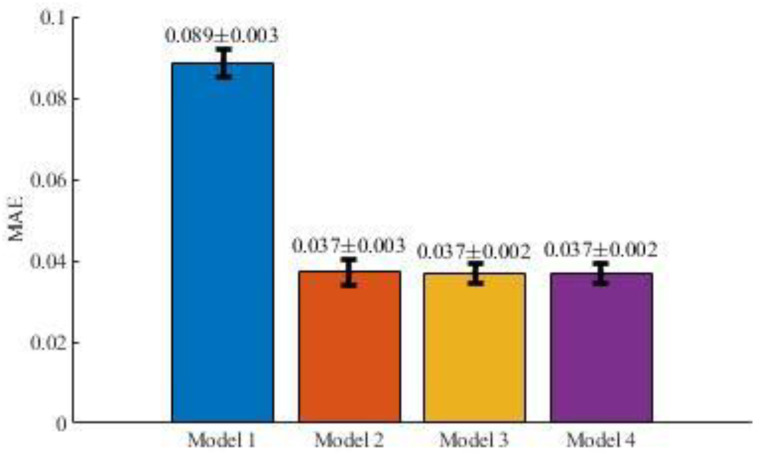
Average MAE metric results and standard deviations after 10-fold cross-validation.

Figure 6 and Table 2 show the average means and standard deviations for the MAE results obtained for the models defined by Equations (2)–(5) in Section 2.3.1. 

**Figure 7:**
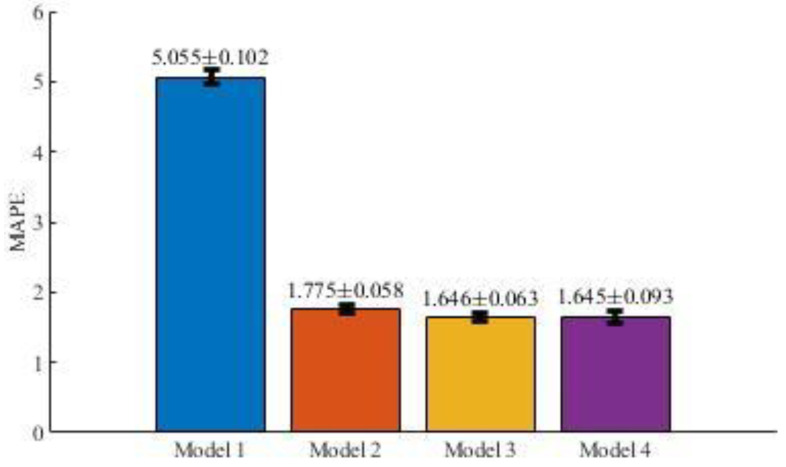
Average MAPE metric results and standard deviations after 10-fold cross-validation.

Analogously, Figure 7 and Table 3 represent the average means and standard deviations for the MAPE results obtained for the models defined by Equations (2)–(5). 

**Figure 8:**
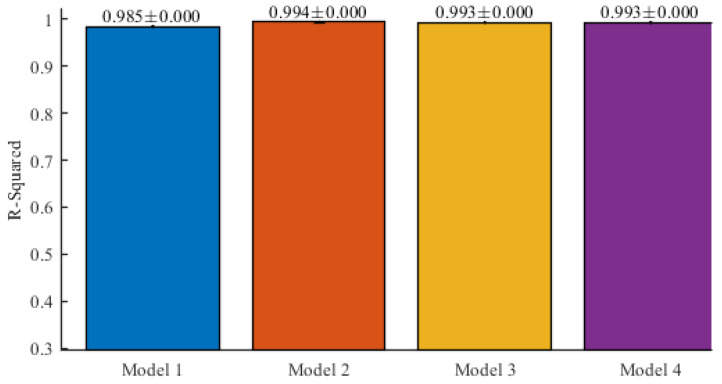
Average R2 metric results and standard deviations after 10-fold cross-validation.

Finally, Figure 8 and Table 4 show the average means and standard deviations for the R-squared results obtained for the models (Model 1, Model 2, Model 3 and Model 4) defined by Equations (2)–(5), respectively. 

**Figure 9:**
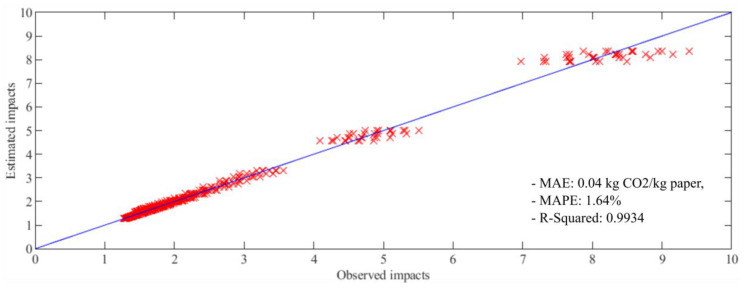
Values of observed impact data and impact data estimated by Model 4.

In Figure 9, the ordinate axis represents the estimations of impact values (measured in kg CO_2_/kg paper) performed by Model 4 (the model which achieved the best performance). The abscissa axis represents, meanwhile, the true values observed for each estimation carried out by the model. As results, interceptions between true values and estimated values are obtained for each sample of the data and are represented as red crosses. The blue line (with a slope of 45 degrees) represents the best possible estimation. A red cross over the blue line means that observed value and estimated value are equal and a perfect match between model and reality has been achieved in that individual estimation. In this figure, it can be seen that estimations of low impacts are better than estimations of impacts with medium and high magnitudes. However, it is possible to check that all cases are well distributed on both sides of the blue line. This is reflected by the low value results of standard deviation as well. The final **β** parameters obtained for each model are presented in Table 5.

## 4. Discussion

Results show clearly that a) the model with the worst performance is Model 1 and, b) the behaviours of the other models are similar to each other. If the first model is not considered, all average values obtained for the MAPE metric are less than 5%. Hence, it can be considered that the performance of models 2, 3 and 4 well represent the behaviour of the response variable (the impact of the circulation of a newspaper) using the selected predictors as inputs.

However, it is interesting to point out that Model 4, defined by Equation (5) in Section 2.3.1, is the most simplified estimator of the environmental impacts. With fewer independent variables, this model achieves similar or even better results than the other non-linear expressions evaluated. This is beneficial for the final results because potential users will depend on less recorded variables to obtain similar performances. The independent contribution of each variable to the estimations can also be assessed. In this sense, analysis of the graphical behaviour and meaning of every independent variable considered and the target variable (environmental impact of the newspaper) provide interesting results: on the one side, the information collected by print run and paper type variables is indispensable for all adjusted non-linear models; on the other, the height of the paper, the grammage and the number of pages can be avoided by a very good adjusted model.

Because the concrete area of study, focused on estimating the environmental impact of a newspaper’s circulation, is unexplored yet, we could not compare our results with other researched models. Nevertheless, as was commented before, average MAPE metrics are below 5%, so the model can be considered appropriately good. Model 4, with a very simplified version of the original non-linear expression, just composed of six β unknown parameters and two independent variables and a MAPE <2 %, is particularly interesting. It enables any press company to easily calculate from this expression the environmental impacts of the newspaper, broken down by each type of product or product specification that the company can manufacture. Additionally, if some new variables are to be incorporated, the procedure outlined in this article can be replicated to obtain similar results.

The specific behaviour of estimations and models evaluated in this article (see Figure 9) invite exploration of the use of some other regression techniques which can structure the information. The discriminatory capacity of some techniques, such as classification and regression trees (CART), based on structured rules [28,29], could be useful for modelling this problem. 

The policies that governments are establishing to minimize climate change will force companies to be aware of the environmental impacts of their activity and to properly and intelligibly incorporate this information into the products they produce.

Based on the LCA of the organization, companies could customize the environmental impacts of their products, either by using an LCA software, (which usually has a high maintenance cost), or by using a simplified methodology like the one proposed in this article, which specifies the impacts for the various possible scenarios.

Moreover, the eco-labelling of products requires information that could be updated and adapted according to the changing production conditions at any time. This information can be provided by the proposed methodology.

## 5. Conclusions

In this article, four non-linear expressions are analysed to model and estimate the environmental impacts (the global warming potential, as a case study) of a printed newspaper for different scenarios and combinations of parameters. The aim of obtaining a correlation that enables the calculation of the environmental impacts for any combination of scenarios without having to have a specific calculation software has been achieved.

All models can estimate low impacts more accurately than medium and high magnitude impacts. However, three of the models (Models 2, 3 and 4) show very satisfactory results over the whole range of application, as their respective parameters have been obtained with a mean absolute percentage error of less than 2%. Not all the independent variables make the same contribution to the estimations: print run and paper type are essential for the proper fit of all non-linear models. However, the contribution of the height of the paper, the grammage and the number of pages is negligible.

It is worth mentioning that Model 4, with just six β unknown parameters and two independent variables (print run and paper type), is particularly accurate (MAPE < 2%) and easy to implement.

In consequence, this paper provides companies in the newspaper production industry with a simple correlation for the estimation of the global warming potential of their products. In addition, the results show that it is possible to have a tool at the disposal of these companies that, based on the historical or environmental impact analysis in different scenarios, would allow them to obtain environmental impacts through correlations in other situations. This methodology could be extended to an endless number of companies and products, so that companies could provide information on the impacts of the products they manufacture that can be part of their environmental labels.

In addition, the different behaviours of the estimations performed by the evaluated models, in relation to the impact magnitudes, invite exploration of the use of some other structured rule-based techniques—classification and regression tree (CART) analysis, for example—for this purpose. 

## Figures and Tables

**Table 1:** Sample of data recorded during the production process.

Row	Pages	Grammage	Height	Paper Type	Print Run	Impact
Number	(#)	(g/m^2^)	(mm)	(-)	(#)	(CO_2_ eq/kg)
1	32	42	360	1	500	9.392
2	32	42	360	1	1000	5.506
3	32	42	360	1	2000	3.563
⋮	⋮	⋮	⋮	⋮	⋮	⋮
1102	32	45	420	2	30000	1.314
1103	32	45	420	2	31000	1.310
1104	32	45	420	2	32000	1.307
⋮	⋮	⋮	⋮	⋮	⋮	⋮
2203	48	42	390	4	9000	1.756
2204	48	42	390	4	10000	1.717
2205	48	42	390	4	11000	1.685
⋮	⋮	⋮	⋮	⋮	⋮	⋮
3304	48	48.8	390	1	39000	1.700
3305	48	48.8	390	1	40000	1.698
3306	48	48.8	390	1	41000	1.696
⋮	⋮	⋮	⋮	⋮	⋮	⋮
4405	64	45	360	3	18000	1.560
4406	64	45	360	3	19000	1.549
4407	64	45	360	3	20000	1.540
⋮	⋮	⋮	⋮	⋮	⋮	⋮
5506	64	48.80	420	4	4800	1.569
5507	64	48.80	420	4	4900	1.568
5508	64	48.80	420	4	5000	1.566

**Table 2:** MAE results for each model in cross-validation.

Iteration	Model 1	Model 2	Model 3	Model 4
1	0.094	0.038	0.036	0.039
2	0.087	0.037	0.038	0.035
3	0.084	0.031	0.037	0.034
4	0.084	0.034	0.036	0.038
5	0.088	0.039	0.035	0.035
6	0.091	0.039	0.043	0.037
7	0.092	0.040	0.038	0.038
8	0.091	0.039	0.037	0.033
9	0.087	0.033	0.036	0.039
10	0.087	0.041	0.034	0.040
*Average*	*0.089*	*0.037*	*0.037*	*0.037*
*Standard deviation*	*0.003*	*0.003*	*0.002*	*0.002*

**Table 3:** MAPE results for each model in cross-validation.

Iteration	Model 1	Model 2	Model 3	Model 4
1	5.106	1.836	1.601	1.640
2	5.193	1.765	1.690	1.590
3	4.836	1.672	1.703	1.570
4	4.990	1.700	1.564	1.689
5	5.033	1.747	1.593	1.615
6	5.157	1.833	1.765	1.613
7	5.015	1.847	1.663	1.757
8	5.087	1.803	1.665	1.478
9	5.012	1.769	1.578	1.793
10	5.118	1.776	1.640	1.703
*Average*	*5.055*	*1.775*	*1.646*	*1.645*
*Standard deviation*	*0.102*	*0.058*	*0.063*	*0.093*

**Table 4:** R-squared results for each model in cross-validation.

Iteration	Model 1	Model 2	Model 3	Model 4
1	0.9848	0.9942	0.9934	0.9933
2	0.9840	0.9939	0.9932	0.9934
3	0.9853	0.9943	0.9930	0.9935
4	0.9851	0.9942	0.9934	0.9936
5	0.9853	0.9941	0.9933	0.9936
6	0.9845	0.9943	0.9935	0.9931
7	0.9843	0.9944	0.9933	0.9933
8	0.9845	0.9942	0.9934	0.9932
9	0.9848	0.9941	0.9938	0.9934
10	0.9848	0.9941	0.9931	0.9932
*Average*	*0.9847*	*0.9942*	*0.9933*	*0.9934*
*Standard deviation*	*0.0004*	*0.0001*	*0.0002*	*0.0002*

**Table 5:** β parameters obtained for each model.

β Parameter	Model 1	Model 2	Model 3	Model 4
β_1_	0.387	−0.377	0.911	0.917
β_2_	0.386	−0.401	0.143	0.142
β_3_	0.018	0.000	0.574	0.573
β_4_	0.405	28.607	−32.838	−35.833
β_5_	−0.088	−1.296	0.000	3280.310
β_6_	0.417	7.487	3287.628	−0.996
β_7_	−0.082	−0.271	−0.996	-
β_8_	0.283	0.142	-	-
β_9_	−45,785,423.699	0.573	-	-
β_10_	3345.275	−7210.433	-	-
β_11_	−0.999	0.000	-	-
β_12_	-	3368.258	-	-
β_13_	-	−1.000	-	-
